# Healthcare-associated *Pneumocystis jirovecii* transmission in the era of universal masking and distancing

**DOI:** 10.1017/ice.2026.10446

**Published:** 2026-06

**Authors:** Florence Durocher, Simon Frédéric Dufresne, Philippe Jean Dufresne, Xavier Marchand-Senécal

**Affiliations:** 1 Department of Microbiology, Infectious Diseases and Immunology, Faculty of Medicine, Université de Montréal, Montréal, QC, Canada; 2 Division of Infectious Diseases and Clinical Microbiology, Department of Medicine, Hôpital Maisonneuve-Rosemont, Montréal, QC, Canada; 3 Maisonneuve-Rosemont Hospital Research Center, Montréal, QC, Canada; 4 Laboratoire de santé publique du Québec, Institut national de santé publique du Québec, Montréal, QC, Canada

**Keywords:** *Pneumocystis jirovecii*, Infection prevention, Healthcare-associated transmission, Universal masking, Airborne transmission

## Abstract

**Objective:**

*Pneumocystis jirovecii* pneumonia is a serious opportunistic infection in immunocompromised individuals. Despite recognized person-to-person transmission and healthcare-associated outbreaks, optimal infection control strategies remain unclear. The COVID-19 pandemic led to the implementation of universal masking and physical distancing in hospitals, providing a unique setting to observe *P. jirovecii* transmission under stringent “droplet precaution”-like conditions. This study investigated healthcare-associated *P. jirovecii* transmission between June 2020 and November 2021.

**Design:**

Retrospective cohort study.

**Setting:**

One tertiary-care hospital in Montréal, QC, Canada.

**Patients:**

All patients with *P. jirovecii* pneumonia at our institution during that period.

**Methods:**

Cases were identified via laboratory data and chart review. *P. jirovecii*-positive samples underwent genotyping using multilocus sequence typing. A transmission map was constructed based on shared genotypes and spatiotemporal overlap of hospital visits within a defined window of potential exposure.

**Results:**

Twenty-eight *P. jirovecii* pneumonia cases were identified. Genotyping succeeded at providing a distinct sequence type (ST) in 21 cases, revealing 7 patients with shared genotypes (3 with ST52, 2 with STX7, 2 with ST19). The transmission map of 12 patients with shared or unknown genotypes revealed 34 same-day and 34 within-one-day contacts, exclusively within outpatient clinics and imaging facilities. Three spatiotemporal clusters of plausible healthcare-associated transmission were identified despite universal masking.

**Conclusion:**

The occurrence of plausible healthcare-associated *P. jirovecii* transmission despite stringent universal masking suggests that traditional “droplet precautions” alone may be insufficient to prevent spread, supporting airborne transmission. Infection prevention strategies may need to be expanded in high-risk settings and should account for airborne transmission.

## Introduction

*Pneumocystis jirovecii* causes life-threatening pneumonia in a variety of immunocompromised hosts. A growing body of experimental and clinical evidence suggests that *P. jirovecii* does not have non-human reservoir and is transmissible from person to person, even causing health care-associated outbreaks.^1,2^ However, despite numerous outbreaks having been investigated and reported in past three decades, the precise modes of transmission remain elusive. Consequently, best infection control and prevention practices are still actively debated.

In the midst of the Coronavirus Disease 2019 (COVID-19) pandemic, our institution, like many others worldwide, has implemented a universal masking policy and physical distancing for all patients and healthcare workers (HCW). This unprecedented infection control measure has provided a unique opportunity to observe healthcare-associated *P. jirovecii* transmission under very stringent and ubiquitous conditions akin to “droplet precaution.” Seeking to further our understanding of in-hospital *P. jirovecii* transmission, we undertook a thorough investigation of all *P. jirovecii* pneumonia cases diagnosed at our institution while the policy was in place. We herein present clinical and molecular epidemiological evidence of active healthcare-associated transmission under these strict precautions.

## Methods

### Ethics

The study was approved by our institution’s ethics committee.

### Study design and population

This is a single-center retrospective cohort study, encompassing all *P. jirovecii* pneumonia cases that have occurred at our institution between June 2020 and November 2021, inclusively. This 18-month study period corresponds to a time when control and prevention measures were the most strictly enforced. *P. jirovecii* cases were identified using our laboratory informatics system by extracting and reviewing all positive *P. jirovecii* tests (direct immunofluorescence assay and nucleic acid amplification tests (NAAT)), as well as all positive serum beta-D-glucan assays. A chart review was next conducted to collect basic demographic and clinical data. Cases were classified according to the EORTC-MSG/ERC criteria.^3^ We included both probable and proven cases, while excluding possible cases that lacked mycological data. Cases considered to represent colonization—i.e., *P. jirovecii* detection without fulfilling the clinical criteria for *P. jirovecii* pneumonia cases—were also included, given the potential for these individuals to contribute to transmission.^4^

### Pneumocystis detection assays

At our center, the main diagnostic test for *P. jirovecii* pneumonia is an immunofluorescence assay (IFA) performed on bronchoalveolar lavage fluid, using the MONOFLUO *Pneumocystis jirovecii* IFA Test Kit (Bio-Rad Laboratories, Inc.). When the IFA result is negative but clinical suspicion remains moderate to high, samples are further tested by nucleic acid amplification testing (NAAT) at our provincial mycology reference laboratory (Laboratoire de santé publique du Québec; LSPQ), using a commercial quantitative PCR assay (RealStar® *Pneumocystis jirovecii* PCR Kit 1.0, Altona Diagnostics, GmbH). Serum beta-D-glucan testing is requested at the discretion of the treating physician, on a case-by-case basis, as an adjunct to diagnosis. This testing is performed at MiraVista Diagnostics (Indianapolis, Indiana), using Fungitell® assay (Associates of Cape Cod, Inc.).

### Genotyping

Genotyping of *P. jirovecii*-positive samples is routinely requested at our institution since 2013, following major outbreaks among renal and stem cell transplant recipients. It is conducted systematically and in real time since 2016 for all *P. jirovecii* pneumonia cases diagnosed in transplant cohorts and in most patients with hematologic malignancies. Additional cases undergo genotyping upon request by treating physicians or the infection prevention and control service. During the study period, genotyping was attempted on all *P. jirovecii* pneumonia cases, except for the few with a very low fungal burden detected by NAAT, where it was associated with amplification failure. Genotyping is performed at LSPQ using ISHAM’s standard four-target (*CYB*, *SOD*, *mt26S*, and *BTUB*) multilocus sequence typing (MLST) method.^5^ Alleles and sequence type were further assessed on the Fungal MLST database, accessible online (https://mlst.mycologylab.org/page/PJPasic2020).

### Transmission map

*P. jirovecii* pneumonia cases involving genotypes shared by at least two patients, as well as those for which genotyping was unavailable (unsuccessfully amplified secondary to a low fungal burden), were included in a transmission network analysis. For selected patients, all hospital visits were retrieved, including inpatient admissions and outpatient visits to clinics, collection centers, and imaging facilities. For each case, visits were documented during a time window reflecting potential incubation and contagiousness, defined as spanning from 180 days before to 30 days after the *P. jirovecii* pneumonia diagnosis. These visits were compiled with a one-day level of granularity. A potential contact was defined as two cases being present at the same location on the same day. However, contacts occurring within a margin of ±1 day (hereafter referred to as one-day contacts) were also recorded. Contacts between two already diagnosed cases were excluded from the analysis. A transmission map was constructed to visually represent potential pathways of transmission between cases.

### Overview of infection control and prevention measures

During the study period, properly worn medical masks were mandatory for all patients, HCW, and visitors. This requirement was enforced by security guards stationed at all restricted hospital entrances, as well as by HCW, including clerical staff. Hospitalized patients were permitted to remove their masks while in their rooms but were strongly encouraged to wear them whenever a HCW entered. HCW were required to wear masks at all times, except when eating in designated, restricted areas without patient contact and while maintaining physical distancing from one another. Importantly, visitor access was significantly restricted, greatly limiting contact between hospitalized patients and outside individuals. While there was a heightened focus on hand hygiene among healthcare workers and the general population during the COVID-19 pandemic (study period), compliance audit data specifically targeting patients were not available. Patients and healthcare providers were not required to wear gloves. Environmental cleaning protocols for outpatient settings (daily cleaning and disinfection with 0.1% sodium hypochlorite) remained unchanged during the study period.

### Softwares

Epidemic curve was produced using Prism 10 (version 10.1.0, GraphPad Software, LLC, 2023). Transmission map was created with Canva (web version, Canva Pty Ltd, 2024).

## Results

### Description of included cases

A total of 28 *P. jirovecii* pneumonia cases were identified during the 18-month study period, including 13 proven, 13 probable, and 2 cases representing colonization. All cases occurred in distinct patients, whose characteristics are outlined in Table 1 and described in further details in Table S1. The population was predominantly female, presented preponderantly with hematological malignancies, or had undergone allogenic hematopoietic stem cell transplant. Most of the patients had no *P. jirovecii* prophylaxis at the time of diagnosis. All cases had a positive NAAT, and most of the samples were collected by bronchoalveolar lavage. For comparison, 84 cases of *P. jirovecii* pneumonia were identified over the 53-month period from January 2016 to May 2020 (corresponding to a mean of 1.55 cases per month, versus 1.58 cases per month during the study period).

**Table 1.** Demographic and clinical characteristics of all *P. jirovecii* pneumonia cases included in the study (n = 28) Table 1 long description.

Characteristics	Cases (%)
*Sex* Female	20 (71.4)
*Mean age* (range)	62,3 (34–81)
*Comorbidities* ^1^ Hematologic malignancies Post-allogenic transplant Solid malignancies Post-autologous transplant Post-CAR T-cells therapy Post-solid organ transplant Rheumatologic conditions HIV DRESS	11 (39.3) 9 (32.1) 2 (7.1) 1 (3.6) 1 (3.6) 1 (3.6) 1 (3.6) 1 (3.6) 1 (3.5)
*P. jirovecii prophylaxis* None Pentamidine TMP-SMX Atovaquone	25 (89.3) 3 (10.7) 0 (0) 0 (0)
*P. jirovecii infection* (according to EORTC-MSG/ERC criteria): Proven Probable Colonization	13 (46.4) 13 (46.4) 2 (7.2)
*Diagnosis of P. jirovecii* pneumonia PCR positive alone Immunofluorescence and PCR positive Immunofluorescence positive alone B-D-glucan positive alone	15 (53.6) 13 (46.4) 0 (0) 0 (0)
*Median number of copies/mL of P. jirovecii PCR* (range)	21, 414 (24–8,673,000)
*Specimen type* Bronchoalveolar lavage Induced sputum Bronchial aspiration	25 (89.3) 2 (7.1) 1 (3.6)
*P. jirovecii genotypes* Unknown Unrelated ST52 STX7 ST19	14 (50.0) 7 (25.0) 3 (10.7) 2 (7.2) 2 (7.1)

1 Mutually exclusive categories, showing the most relevant or recent predisposing condition.

CAR T-cells, chimeric antigen receptor T-cells; HIV, human immunodeficiency virus; DRESS, drug reaction with eosinophilia and systemic symptoms.

**Figure 1.**
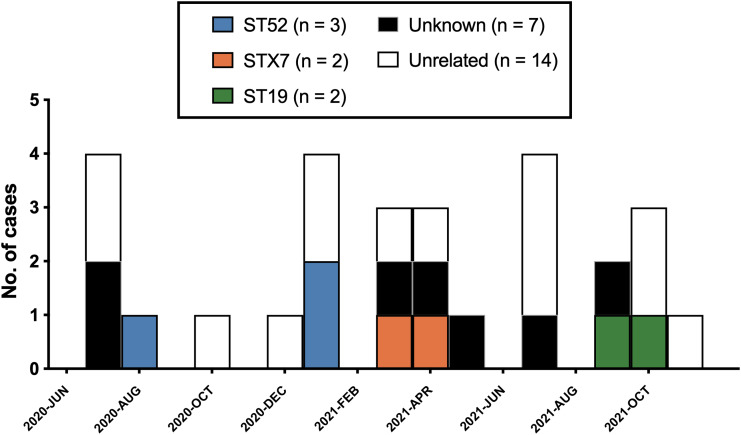
Figure 1 long description. Epidemic curve of all *P. jirovecii* pneumonia cases included in the study (n = 28).

Genotyping was attempted in 24 of 28 cases; the remaining samples had signals too weak to be successfully amplified. It succeeded at providing a distinct sequence type in 21 (in 75% of all patients or 88% of patients with sufficient burden). Among the successfully genotyped cases (n = 21), 14 (67%) represented unrelated genotypes. In contrast, 7 cases (33%) involved sequence types that were shared by at least one other patient (3 patients with ST52, 2 with STX7, and 2 with ST19). An epidemic curve showing *P. jirovecii* pneumonia cases and their respective sequence types illustrates temporal clustering of genetically related cases (Figure 1).

### Transmission map

**Figure 2.**
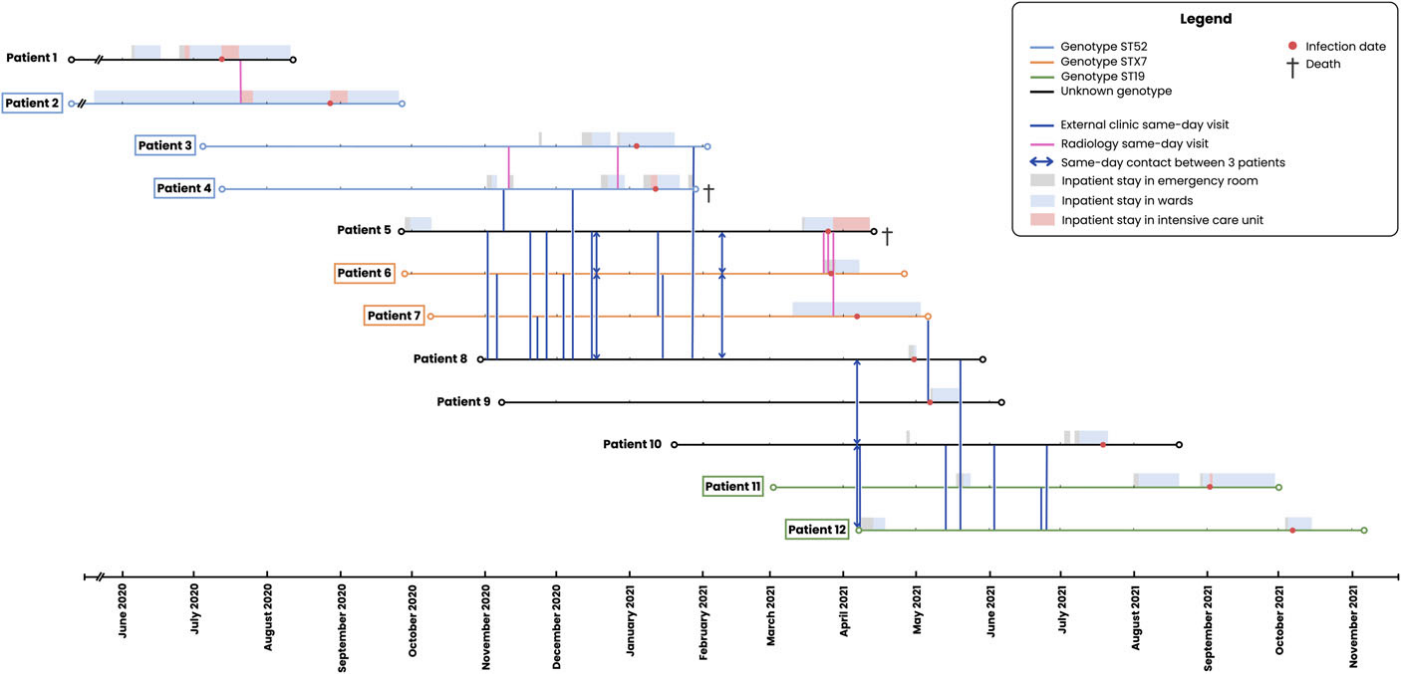
Figure 2 long description. Transmission map for *P. jirovecii* pneumonia cases with shared or unknown genotypes (n = 12). **Legend:** Map of potential transmission events between patients infected or colonized by the fungus. The y-axis displays 12 individual patients categorized by shared (colored lines) or unknown genotypes (black lines); the x-axis and horizontal bars represent outpatient visits and inpatient stay histories. Based on the study definition of contact for possible transmission, 34 same-day encounters were identified (vertical lines). Vertical lines with arrowheads indicate same-day contact involving 3 patients (equivalent to 3 distinct contacts). For clarity, one-day contacts are not shown in the Figure. All identified contacts occurred exclusively in outpatient settings or imaging facilities. Although Patient 2 shares the same genotype as Patient 3 and 4 (ST52), no contact was identified, suggesting the presence of missing links (asymptomatic carriers).

Patients with either shared or unknown genotypes underwent a thorough transmission analysis. A total of 12 patients were included in the transmission map—7 with shared genotypes and 5 with unknown genotypes (Figure 2). Two patients with unknown genotypes were excluded from the map because they had no potential contact with any other case.

Only contacts between patients potentially sharing the same genotype were recorded in the transmission map. Accordingly, we included contacts between (a) two patients sharing a known genotype, (b) two patients with unknown genotypes, and (c) one patient with a known genotype and another with an unknown genotype. Based on these criteria, we identified a total of 34 same-day contacts and 34 one-day contacts, acknowledging that some may not represent true transmission of the same genotype. All these contacts occurred within radiology facilities or during outpatient clinic visits, including both medical consultations and treatment administrations. No contacts occurred in inpatient settings.

The map revealed plausible healthcare-associated transmission in most cases. Patients infected with the ST52 genotype had several hospital contacts, particularly within imaging facilities. Some hospitalization episodes also overlapped—most notably between Patients 3 and 4—although no concurrent presence within the same hospital floor or unit was identified for any patients. No direct link could be established between Patient 2 and the other two patients with the same genotype, suggesting a role for asymptomatic hosts not captured by this study in the transmission chain. Patients infected with the STX7 genotype (a novel genotype, not previously described) displayed limited direct contact with each other but shared multiple significant contacts with Patients 5 and 8, both of whom had unknown genotypes—suggesting potential healthcare-associated transmission within this cluster. A similar pattern was observed for the ST19 genotype: although there were few direct contacts between the two patients with confirmed ST19, possible epidemiological links were identified with Patients 9 and 10, both of unknown genotype.

Notably, demographic data revealed that most patients within the same clusters resided in different administrative regions at the time of their *P. jirovecii* pneumonia episodes (data not shown). Furthermore, the STX7 and ST19 genotypes had never been identified in our institution among the 58 patients whose strains were genotyped between 2016 and the study period. Altogether, these findings suggest that community-based transmission of a locally prevalent genotype is unlikely.

## Discussion

In this study—enabled by the unique circumstances of a concurrent pandemic involving a respiratory virus—we observed plausible *P. jirovecii* transmission occurring despite the implementation of a stringent universal masking policy. The identification of three spatiotemporal clusters of plausible healthcare-associated transmission during this period suggests that traditional “droplet precautions” using medical masks may be insufficient to fully prevent disease spread. This supports the likelihood of airborne transmission of infectious respiratory particles, rather than “direct deposition,” as defined by the recently revised World Health Organization terminology for airborne pathogen transmission.^6^ Our findings add on both experimental and observational data in animals and humans that support the airborne dissemination of *P. jirovecii.* ^1,2^ Recognizing the potential for airborne transmission has implications for developing more effective prevention strategies for *P. jirovecii* pneumonia in at-risk patients. Currently, most clinical practice guidelines for infection control and prevention of *P. jirovecii* pneumonia recommend only that patients with *P. jirovecii* pneumonia not share a room with other vulnerable individuals.^7,8^ In the context of an outbreak, reported interventions primarily focus on enhancing prophylaxis for at-risk patients. Hence, robust evidence to guide infection control and prevention practices for *P. jirovecii* pneumonia is still needed.

The role of asymptomatic *P. jirovecii* carrier (HCW or patient) was not specifically assessed in this study on transmission. However, despite rigorous efforts to establish epidemiological links between cases to construct a transmission map, a proportion of cases lacked clear connections indicative of direct transmission, suggesting the potential presence of missing links within the *P. jirovecii* transmission chain. Experimental and epidemiological evidence already demonstrate that asymptomatic hosts, like colonized HCW or patients, can serve as reservoirs and transmit *P. jirevocii* to susceptible hosts,^9–11^ even serving as index cases in clusters.^4^ The implication of these observations is that infection prevention strategies should extend beyond symptomatic individuals to effectively mitigate the spread of this opportunistic infection. This consideration echoes a central aim of the universal masking policy enacted during the COVID-19 pandemic, which sought to limit SARS-CoV-2 transmission from asymptomatic individuals.

Although plausible transmission of *P. jirovecii* was observed during a period when a universal masking policy was in place, this does not imply that medical masks are entirely ineffective in preventing it. Under such policies, medical masks likely offer dual benefits: providing some respiratory protection to the wearer and serving as source control by containing exhaled infectious respiratory particles. It is plausible that masks contribute to a reduction in *P. jirovecii* transmission—a phenomenon observed with other airborne pathogens. For instance, mask use by individuals with tuberculosis has been shown to reduce transmission by approximately 50%.^12^ While the present study did not quantitatively assess the impact of masking and physical distancing policies on *P. jirovecii* transmission, other observational studies have reported a marked decrease in *P. jirovecii* pneumonia incidence following the implementation of nationwide non-pharmaceutical interventions, including masking, during the early stages of the COVID-19 pandemic.^13^

This study has several limitations. First, the transmission map illustrates opportunities for contact but does not provide definitive proof of transmission. Moreover, genotyping data was unknown for seven patients, limiting our ability to accurately evaluate the transmission dynamics. MLST, while useful, is not the most sensitive technique, particularly for detecting mixed infections with multiple genotypes. Although its discriminatory power is imperfect and identical genotypes may cluster by chance alone, the Hunter-Gaston diversity index was calculated at 0.975—well above the threshold typically required for a reliable MLST typing scheme.^5^ Nevertheless, the clustering of similar sequence types, the presence of uncommon genotypes, and the patients’ residence in geographically distant regions collectively support the likelihood of healthcare-associated acquisition and lend plausibility to the proposed routes of transmission.

While data on air exchange rates in rooms and audit on masking, hand hygiene, and environmental cleaning practices were not captured, several factors mitigate this limitation.

Lax implementation of the universal masking policy seems improbable, given the strict enforcement strategies in place at the time. Also, current literature identifies contaminated air as the primary vector for *P. jirovecii*, with no evidence supporting transmission via water, food, or environmental fomites.^2,14,15^ Consequently, the role of hand hygiene and surface cleaning in preventing transmission is likely limited in this context.

Furthermore, the absence of precise pre and postmasking comparative data prevents quantitative evaluation of the impact of this infection prevention measure on the incidence of healthcare-associated *P. jirovecii* pneumonia. We elected not to use a pre/post study design because several factors hindered its feasibility. First, a local policy requiring kidney and hematopoietic stem cell transplant patients to wear masks in waiting rooms had been in effect since a *P. jirovecii* pneumonia outbreak years before the study period, thereby attenuating differences between the pre and postintervention periods. Second, important concomitant changes occurred during the study period, notably the widespread adoption of non-pharmacological interventions for COVID-19 prevention in the community, which could have significantly influenced overall case incidence, as reported in Korean data.^13^

Finally, other inherent limitations to *P. jirovecii* pneumonia remain, notably the lack of clear knowledge regarding its incubation period and contagiousness, which limits the interpretation of temporal and spatial exposure patterns. In our study, a 180-day window was elected, but it is possibly an overestimation since diagnosis of cases in a cluster are usually only separated by days or weeks.

Recognizing limitations, we believe that our data strongly supports an airborne transmission of *P. jirovecii*, rendering “droplet precautions” against “direct deposition” insufficient for stopping the transmission. Establishing efficient prevention strategies is a priority in this field as *P. jirovecii* pneumonia poses a serious threat for immunocompromised patients. Further studies are needed to better understand transmission and evaluate the efficacy of other interventions such as improved ventilation, negative-pressure rooms (or at least single-patient room), N95 respirators, and the benefit of possibly continuing universal masking policy in wards, clinics, and waiting rooms with patients vulnerable to *P. jirovecii* pneumonia.

## Supporting information

10.1017/ice.2026.10446.sm001 Durocher et al. supplementary material Durocher et al. supplementary material

## Data Availability

The authors confirm that the data supporting the findings of this study are available within the article. Additional data are available from the corresponding author, X.M.S., upon reasonable request.

## Supplementary material

The supplementary material for this article can be found at https://doi.org/10.1017/ice.2026.10446.

## Figure 1 Long description

The bar graph compares the number of Pneumocystis jirovecii pneumonia cases over time, categorized by different strains. The x-axis represents time periods from June 2020 to October 2021, while the y-axis represents the number of cases, ranging from 0 to 5. The graph features vertical, grouped bars for each time period, with each group containing multiple bars representing different strains: ST52 in blue, STX7 in orange, ST19 in green, Unknown in black, and Unrelated in white. The legend indicates the number of cases for each strain. Notable trends include peaks in cases in August 2020, February 2021, June 2021, and August 2021. The Unknown strain has the highest number of cases, followed by Unrelated, ST52, STX7, and ST19. All values are approximated.

Navigate back to Figure 1.

## Figure 2 Long description

A transmission map displays potential transmission events between 12 patients infected or colonized by the fungus Pneumocystis jirovecii. The y-axis lists the 12 patients categorized by shared genotypes using colored lines or unknown genotypes using black lines. The x-axis and horizontal bars represent outpatient visits and inpatient stay histories. Vertical lines indicate same-day encounters, with arrowheads showing same-day contact involving three patients. All identified contacts occur in outpatient settings or imaging facilities. Patient 2 shares the same genotype as Patients 3 and 4 but has no identified contact, suggesting missing links such as asymptomatic carriers.

Navigate back to Figure 2.

## Table 1 Long description

The table presents demographic and clinical characteristics of 28 P. jirovecii pneumonia cases identified during an 18-month study period. It includes data on sex, mean age, comorbidities, P. jirovecii prophylaxis, infection criteria, diagnosis methods, median number of copies per milliliter of P. jirovecii PCR, specimen type, and P. jirovecii genotypes. The table has 15 rows and 2 columns. The first column lists characteristics such as sex, mean age, comorbidities, prophylaxis, infection criteria, diagnosis methods, median number of copies per milliliter of P. jirovecii PCR, specimen type, and P. jirovecii genotypes. The second column provides the corresponding cases or percentages. Notable trends include a predominantly female population, a high prevalence of hematological malignancies or allogenic hematopoietic stem cell transplants, and most patients having no P. jirovecii prophylaxis at the time of diagnosis. All cases had a positive nucleic acid amplification test, and most samples were collected by bronchoalveolar lavage.

Navigate back to Table 1.
