# Supplementary material for: Healthcare-associated Pneumocystis jirovecii transmission in the era of universal masking and distancing
Source: Infect Control Hosp Epidemiol. 2026 Apr 13;47(6):593–8. doi: 10.1017/ice.2026.10446 (PMC13216789; doi:10.1017/ice.2026.10446)
Supplement: Durocher et al. supplementary material [file S0899823X26104462sup001.docx]

**Table S1.** Detailed characteristics of individual *P. jirovecii* pneumonia cases included in the study (n=28).

| **Case (patient number as appearing in Figure 2)** | **Date of Diagnosis** | **Specimen for mycological diagnosis** | **IF result** | **PCR result (copies/mL)** | **Sequence type (ST)** | **Comorbidities^†^** | **Prophylaxis at time of diagnosis** | **Treatment** |
| --- | --- | --- | --- | --- | --- | --- | --- | --- |
| 1 | 07/2020 | BAL | Negative | 1269 | Unrelated | HM | None | CP |
| 2 | 07/2020 | Bronchial aspiration | Positive | 335457 | Unrelated | HSCT | None | TMP-SMX |
| 3 (Patient 1) | 07/2020 | BAL | Negative | 207 | Unknown | HSCT | IP | TMP-SMX, then CP |
| 4 | 07/2020 | BAL | Negative | 16212 | Unknown | KT | None | CP, then Atovaquone |
| 5 (Patient 2) | 08/2020 | BAL | Positive | 146475 | ST52 | DRESS | None | CP |
| 6 | 10/2020 | BAL | Positive | 4884 | Unrelated | HM | None | TMP-SMX |
| 7 | 12/2020 | BAL | Positive | 64323 | Unrelated | HM | None | TMP-SMX |
| 8 (Patient 3) | 01/2021 | BAL | Negative | 133179 | ST52 | HM | None | TMP-SMX |
| 9 (Patient 4) | 01/2021 | BAL | Positive | 450000 | ST52 | Solid malignancy | None | TMP-SMX |
| 10 | 01/2021 | BAL | Negative | 31206 | Unrelated | HM | None | TMP-SMX |
| 11 | 01/2021 | BAL | Positive | 8673000 | Unrelated | HM | None | TMP-SMX |
| 12 | 03/2021 | BAL | Positive | 32469 | Unrelated | HSCT | IP | TMP-SMX |
| 13 (Patient 5) | 03/2021 | BAL | Negative | 381 | Unknown | HSCT | None | TMP-SMX |
| 14 (Patient 6) | 03/2021 | BAL | Negative | 2646 | ST-X7* | HSCT | None | TMP-SMX |
| 15 (Patient 7) | 04/2021 | BAL | Negative | 37656 | ST-X7* | HSCT | None | No treatment |
| 16 | 04/2021 | BAL | Negative | 2883 | Unrelated | CAR T-cells | None | TMP-SMX |
| 17 (Patient 8) | 04/2021 | BAL | Positive | 24 | Unknown | HSCT | IP | TMP-SMX |
| 18 (Patient 9) | 05/2021 | BAL | Negative | 156 | Unknown | HM | None | No treatment |
| 19 | 07/2021 | BAL | Positive | 6096000 | Unrelated | HIV | None | TMP-SMX |
| 20 (Patient 10) | 07/2021 | BAL | Negative | 616 | Unknown | HM | None | TMP-SMX |
| 21 | 07/2021 | BAL | Negative | 2985 | Unrelated | HM | None | TMP-SMX |
| 22 | 07/2021 | BAL | Negative | 54105 | Unrelated | HSCT | None | TMP-SMX |
| 23 (Patient 11) | 09/2021 | BAL | Positive | 134100 | ST19 | HM | None | TMP-SMX |
| 24 | 09/2021 | IS | Negative | 1293 | Unknown | RA | None | TMP-SMX |
| 25 (Patient 12) | 10/2021 | BAL | Positive | 24918 | ST19 | HSCT | None | TMP-SMX |
| 26 | 10/2021 | BAL | Positive | 1944000 | Unrelated | HSCT | None | TMP-SMX |
| 27 | 10/2021 | BAL | Positive | 17910 | Unrelated | HM | None | TMP-SMX |
| 28 | 11/2021 | IS | Not done | 5313 | Unrelated | Solid malignancy | None | TMP-SMX |

Abbreviations: BAL: Bronchoalveolar lavage; CAR T-cells, chimeric antigen receptor T-cells; CP: Clindamycin and primaquine; DRESS: drug reaction with eosinophilia and systemic symptoms; HIV: Human immunodeficiency virus; HM: Hematological malignancy; HSCT: Hematopoietic stem cell transplantation; IF: Immunofluorescence; IP: inhaled pentamidine; IS: induced sputum; KT: Kidney transplantation; RA: Rheumatoid arthritis; ST: Sequence type; TMP-SMX: Trimethoprim-Sulfamethoxazole

^†^ Mutually exclusive categories, showing the most relevant or recent predisposing condition.

*ST-X are new, not yet described sequence types (with known alleles)
